# Relationship between TGF-β1 + 869 T/C and + 915 G/C gene polymorphism and risk of acute rejection in renal transplantation recipients

**DOI:** 10.1186/s12881-019-0847-2

**Published:** 2019-06-25

**Authors:** Hong-Yan Li, Tianbiao Zhou, Shujun Lin, Wenshan Lin

**Affiliations:** 1 Department of Nephrology, Huadu District People’s Hospital of Guangzhou, Huadu Hospital of Southern Medical University, 510800, No 22 Baohua Road, Guangzhou, China; 2 Department of Nephrology, the Second Affiliated Hospital of Shantou University Medical College, Shantou, 515041 China

**Keywords:** Acute rejection, Renal transplantation, TGF-β1, Polymorphisms, Meta-analysis

## Abstract

**Background:**

This meta-analysis was conducted to assess the relationship between the transforming growth factor-beta 1 (TGF-β1) + 869 T/C gene polymorphism, + 915 G/C gene polymorphism, and the susceptibility of acute rejection in the recipients with renal transplantation.

**Methods:**

Relevant studies were searched and identified from the Cochrane Library and PubMed, and eligible investigations were recruited and data were calculated by meta-analysis.

**Results:**

In this study, we found no relationship between either TGF-β1 + 869 T/C or TGF-β1 + 915 G/C gene polymorphism and acute rejection susceptibility in patients with renal transplantation. No association between either gene polymorphism and acute rejection susceptibility in patients with renal transplantation in Caucasian, Asian, or African populations individually was found.

**Conclusion:**

The TGF-β1 + 869 T/C and + 915 G/C gene polymorphisms are not associated with acute rejection susceptibility in recipients with renal transplantation.

## Background

End-stage renal disease (ESRD) has been defined as the start of renal replacement therapy or death relegated to renal diseases, its increasing worldwide prevalence represents a major economic and health burden [1, 2]. Renal transplantation is currently the therapy of choice for ESRD in children and adolescents [3, 4]. Approximately 20% of cases of renal disease progress to ESRD, for which the treatment of choice is also a kidney transplant [5]. Acute rejection in patients with renal transplantation can damage the transplanted renal tissue, even to the point of loss of renal function, which can threaten the life of the patient. At present, some studies show that some gene polymorphisms were associated with the risk of acute kidney allograft rejection [6–9], but some studies indicate that some gene polymorphisms were not associated with the risk of acute rejection susceptibility in recipients with renal transplantation [10–12]. Gene polymorphism can affect the expression level and the protein function, and there are some meta-analyses to assess the relationship between some gene polymorphism and the risk of acute rejection of kidney, but the conclusion is conflicting [11, 13–16]. Early detection and accurate management of acute rejection of kidney are important to the long-term health of each transplant recipient [17]. It is essential and very important to study the risk factors for the acute rejection in patients with renal transplantation.

Transforming-growth factor β1 (TGF-β1) is a multifunctional pro-fibrotic cytokine and involves in the physiological processes associated with growth, differentiation, and fibrosis [18, 19]. In contrast, TGF-β1 is a powerful immunoregulatory cytokine that inhibits T-cell activation, and TGF-β1 gene polymorphisms, especially in the position of + 869 T/C or + 915 G/C, encodes the signaling sequence of the TGF-β1 protein, and modify the production of cytokine [20, 21]. TGF-β1 + 869 T/C gene polymorphism results in the change of codon 10 from leucine (T) to proline (C), and TGF-β1 + 915 G/C gene polymorphism results in the change of codon 25 from arginine (G) to proline (C). In vitro, the presence of leucine or arginine, respectively, has been indicated to lead to a higher production of TGF-β1 [22]. TGF-β1 production might correlate with reduced incidence of acute rejection, since it down-regulates Th1 responses and Th1 cytokine production [21]. Single-nucleotide polymorphisms are associated with the risk of some diseases and drug dose requirement in kidney recipients [23–27]. The current evidence indicates that TGF-β1 involves in the pathogenesis of acute rejection in patients with renal transplantation. TGF-β1 + 869 T/C and + 915 G/C gene polymorphisms, which are important variants of TGF-β1, are reported to be associated with the risk of acute rejection.

In previous, Ge et al. [28] investigated the associations between the TGF-β1 polymorphisms of acute rejection susceptibility. It showed that TGF-β1 + 869 T/C gene polymorphism was not associated with the susceptibility of acute rejection in recipients with renal transplantation. Another meta-analysis [29] including 9 studies had investigated the combined effects of human + 869 T/C and + 915 G/C polymorphisms in the TGF-β1 gene with risk factors of renal transplantation, and indicated that recipient TGF-β1 high producer haplotypes were not significantly associated with an increased risk for acute rejection susceptibility in recipients with renal transplantation. However, the sample size is relatively small that it may be omit a small effect. Different types of ethnicity may also lead to different findings. We performed a meta-analysis of more studies to determine whether the TGF-β1 + 869 T/C and + 915 G/C gene polymorphisms are associated with the risk of acute rejection in renal transplantation.

## Methods

### 1. Search strategy

Two investigators (HYL and TBZ) independently searched the Cochrane Library and PubMed databases through October 1, 2018 using the terms ‘(transforming growth factor-beta 1 OR TGF-β1) AND (polymorphism OR genotype OR allele) AND (acute rejection OR early graft rejection OR kidney transplant OR renal transplant OR allograft nephropathy OR rejection graft)’. The references of retrieved reports and association reviews were checked for additional missing data that we failed to identify during the electronic search.

### 2. Inclusion and exclusion criteria

**Inclusion criteria:** (1) The study provided detailed genotype data regarding TGF-β1 + 869 T/C and + 915 G/C distribution; (2) the study was given a case-control design; (3) the outcome was risk of acute rejection in the recipients with renal transplantation.

### Exclusion criteria

(1) study was unrelated to the association between TGF-β1 + 869 T/C and + 915 G/C gene polymorphism and the risk of acute rejection in the recipients with renal transplantation; (2) review articles, case reports and editorials; (3) study had not provided the data of control group; (4) data was incomplete or missing; (5) animal study; (6) data was duplicated.

Two investigators (HYL and TBZ) independently conducted the literature screening process according to inclusion and exclusion criteria, and disagreements were resolved through discussion (WSL and SJL).

### 3. Data extraction

The information was extracted by two investigators (HYL and TBZ) independently from each included study: first author, publication years, country/district of study, ethnicity, and the number of case group and control group for TGF-β1 + 869 T/C and + 915 G/C genotypes. The frequencies of T and G alleles in TGF-β1 + 869 T/C and + 915 G/C were counted for the cases and controls. Disagreements were resolved through discussion (WSL and SJL).

### 4. Quality assessment of the included studies

The quality assessment of the included case-control studies recruited into this study was assessed by 2 investigators (TBZ and HYL) using the method of Newcastle-Ottawa Scale (NOS) [30, 31]. Major aspects to be assessed including selection of study subjects (four scores in total); exposure factors or outcomes (three scores in total); inter-group comparability (two scores in total). A score equal to or higher than 6 was regarded as high-quality studies, otherwise, less than 5 was considered as low-quality studies. Disagreements were resolved through discussion (WSL and SJL).

### 5. Statistical analysis

The pooled OR (odds ratio) with 95% confidence interval (95% CI) were evaluated to test the strength of associations between the TGF-β1 + 869 T/C or + 915 G/C gene polymorphisms and the risk of acute rejection in the recipients with renal transplantation. The I^2^ index was used to check heterogeneity assumption. When I^2^ ≥ 50% and *P* < 0.05, a Der-Simonian and Laird random-effects model was used to analyze data. Otherwise, a Mantel-Haenszel fixed-effects model was used. The Egger regression asymmetry test [32] and Begg adjusted rank correlation test [33] were used to calculate publication bias (*P* < 0.1 was considered significant). The available data from each investigation were extracted and calculated using Cochrane Review Manager Version 5.3 [34]. Hardy-Weinberg equilibrium (HWE) teat was assess for the genotype distribution of the control group (HWE; *P* < 0.05 was considered significant). Sensitivity analysis was performed when studies with controls not in HWE. Sensitivity analysis was also conducted according to omit each individual study and switching from fixed effect to random effect.

## Results

### Study characteristics

**Fig. 1:**
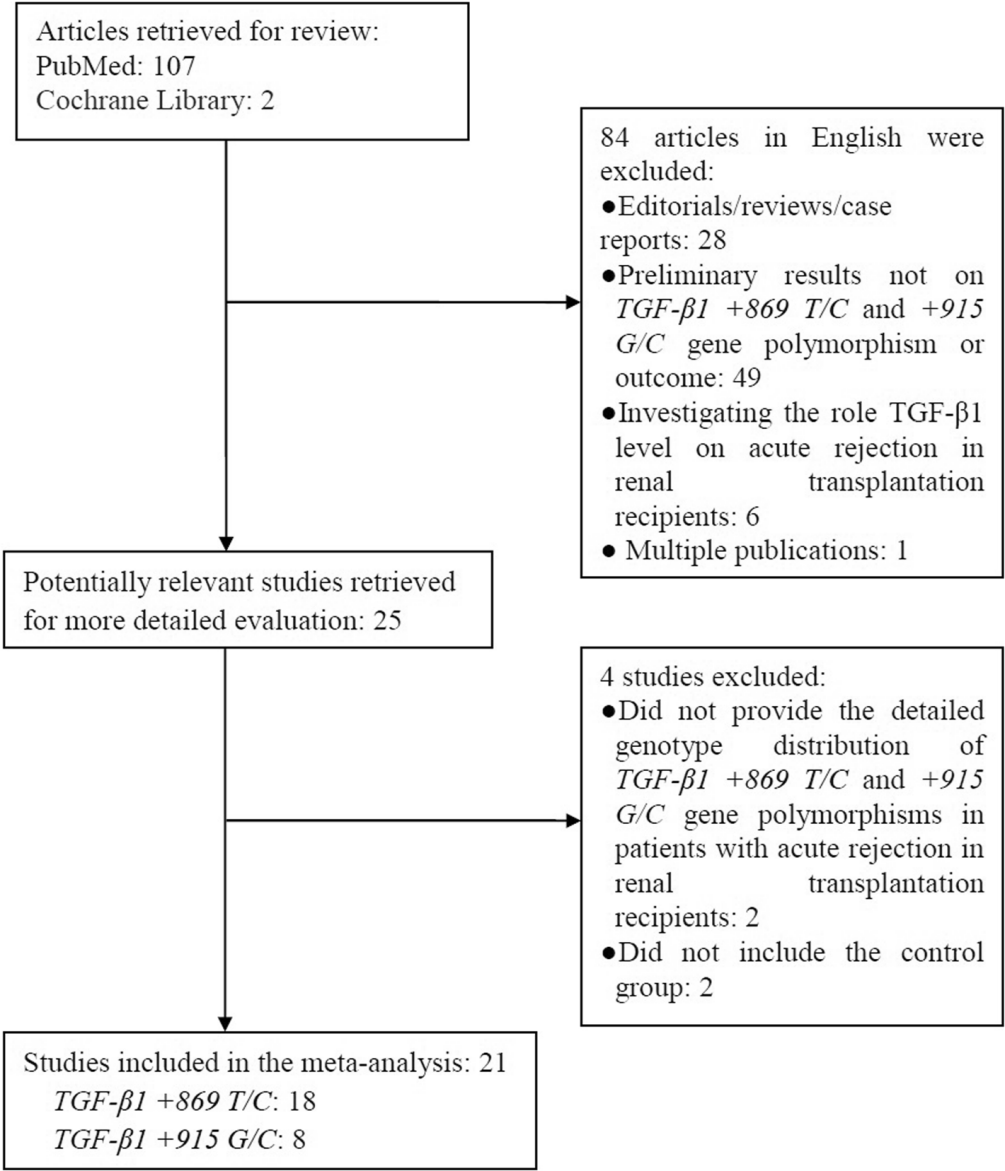
Flow diagram of this meta-analysis

There were 18 studies [20, 35–51] about the relationship between TGF-β1 + 869 T/C gene polymorphism and risk of acute rejection in patients with renal transplant (Fig. 1). We extracted the interesting data and calculated the T allele frequencies of TGF-β1 + 869 T/C in the case group and control group. One thousand five hundred eight patients with acute rejection and 2784 controls were included. Basical characteristics of included studies were presented in Table 1. We used the method of NOS to assess the quality of individual studies, and found all the included studies for TGF-β1 + 869 T/C gene polymorphism was regarded as high-quality studies (Table 1).

**Table 1:** Characteristics of studies evaluating the effects of the transforming growth factor-beta 1 (TGF-β1) + 869 T/C gene polymorphism on the risk of acute rejection in renal transplantation patients

First author and	Country/District	Ethnicity		AR				Non-AR			HWE	NOS
year			TT	TC	CC	Total	TT	TC	CC	Total	(Y/N)	scores
Marshall et al. 2000	UK	Caucasian	46	55	13	114	39	48	8	95	Y	8
Alakulppi et al. 2004	Finland	Caucasian	31	–	–	50	115	–	–	241	–	6
Ligeiro et al. 2004	Portugal	Caucasian	12	12	7	31	14	15	6	35	Y	7
Chow et al. 2005	China	Asian	8	–	–	52	13	–	–	77	–	8
Guo et al. 2005	China	Asian	18	15	6	39	18	57	15	90	N	6
Dmitrienko et al. 2005	Canada	Caucasian	16	24	10	50	16	24	10	50	Y	7
Gendzekhadze et al. 2006	Venezuela	Mixed	12	12	6	30	10	14	9	33	Y	8
Hueso et al. 2006	Spain	Caucasian	6	5	3	14	20	28	15	63	Y	6
Canossi et al. 2007	Italy	Caucasian	4	15	6	25	14	29	18	61	Y	7
Brabcova et al. 2007	Czech Republic	Caucasian	32	91	67	190	34	128	84	246	Y	7
Manchanda et al. 2008	India	Asian	1	11	6	18	19	45	18	82	Y	6
Mendoza-Carrera et al. 2008	México	Mixed	–	–	8	19	–	–	7	32	–	7
Grinyóet al 2008	Spain	Caucasian	18	34	11	63	66	69	26	161	Y	6
Karimi et al. 2012	Iran	Asian	5	8	16	29	17	24	30	71	N	7
Seyhun et al. 2012	Turkey	Caucasian	6	10	3	19	16	31	24	71	Y	7
Dhaouadi et al. 2013	Tunisia	African	55	17	8	80	105	38	8	151	Y	7
Saigo et al. 2014	Japan	Asian	5	16	3	24	22	61	38	121	Y	7
Seyhun et al. 2015	Turkey	Caucasian	6	15	7	28	16	28	18	62	Y	7

*AR* acute rejection, *Non-AR* non- acute rejection, *HWE* Hardy-Weinberg equilibrium, *Y* yes, *N* no, *NOS* Newcastle–Ottawa scale. A total score of NOS for each study can vary from 0 (worst) to 9 (best), low-quality studies: 0 to 4 points; high-quality studies: 5 to 9 points

Eight studies [20, 36, 42 49, 52–55] about the association of TGF-β1 + 915 G/C gene polymorphism with the susceptibility of acute rejection in patients with renal transplantation were included (Fig. 1). The interesting data were extracted, and the G allele frequencies of TGF-β1 + 915 G/C for the case group and the control group were counted. Four hundred sixty-two patients with acute rejection and 1099 controls were included in the 8 studies. The characteristics of included investigations were showed in Table 2. The method of NOS was used to assess the quality of individual studies, and the results indicated that all the included studies for TGF-β1 + 915 G/C gene polymorphism was regarded as high-quality studies (Table 2).

**Table 2:** Characteristics of the studies evaluating the effects of the transforming growth factor-beta 1 (TGF-β1) +915 G/C gene polymorphism on the risk of acute rejection in renal transplantation patients

First author and	Country/District	Ethnicity		AR				Non-AR			HWE	NOS
year			TT	TC	CC	Total	TT	TC	CC	Total	(Y/N)	scores
Alakulppi et al. 2004	Finland	Caucasian	48	–	–	50	210	–	–	241	–	6
Park et al. 2004	Korea	Asian	13	0	0	13	151	0	0	151	Y	7
Dmitrienko et al. 2005	Canada	Caucasian	40	8	2	50	43	6	1	50	N	7
Li et al. 2007	China	Asian	40	6	4	50	49	15	6	70	Y	8
Seyhun et al. 2012	Turkey	Caucasian	17	1	1	19	48	23	0	71	Y	7
Dhaouadi et al. 2013	Tunisia	African	55	17	8	80	105	38	8	151	Y	7
Chen et al. 2014	France etc.	Caucasian	41	9	0	50	243	31	1	275	Y	7
Seyhun et al. 2015	Turkey	Caucasian	120	28	2	150	66	23	1	90	Y	7

*AR* acute rejection, *Non-AR* non- acute rejection, *HWE* Hardy-Weinberg equilibrium, *Y* yes, *N* no, *NOS* Newcastle–Ottawa scale. A total score of NOS for each study can vary from 0 (worst) to 9 (best), low-quality studies: 0 to 4 points; high-quality studies: 5 to 9 points

### Association of TGF-β1 + 869 T/C gene polymorphism with the susceptibility of acute rejection in patients with renal transplantation

**Fig. 2:**
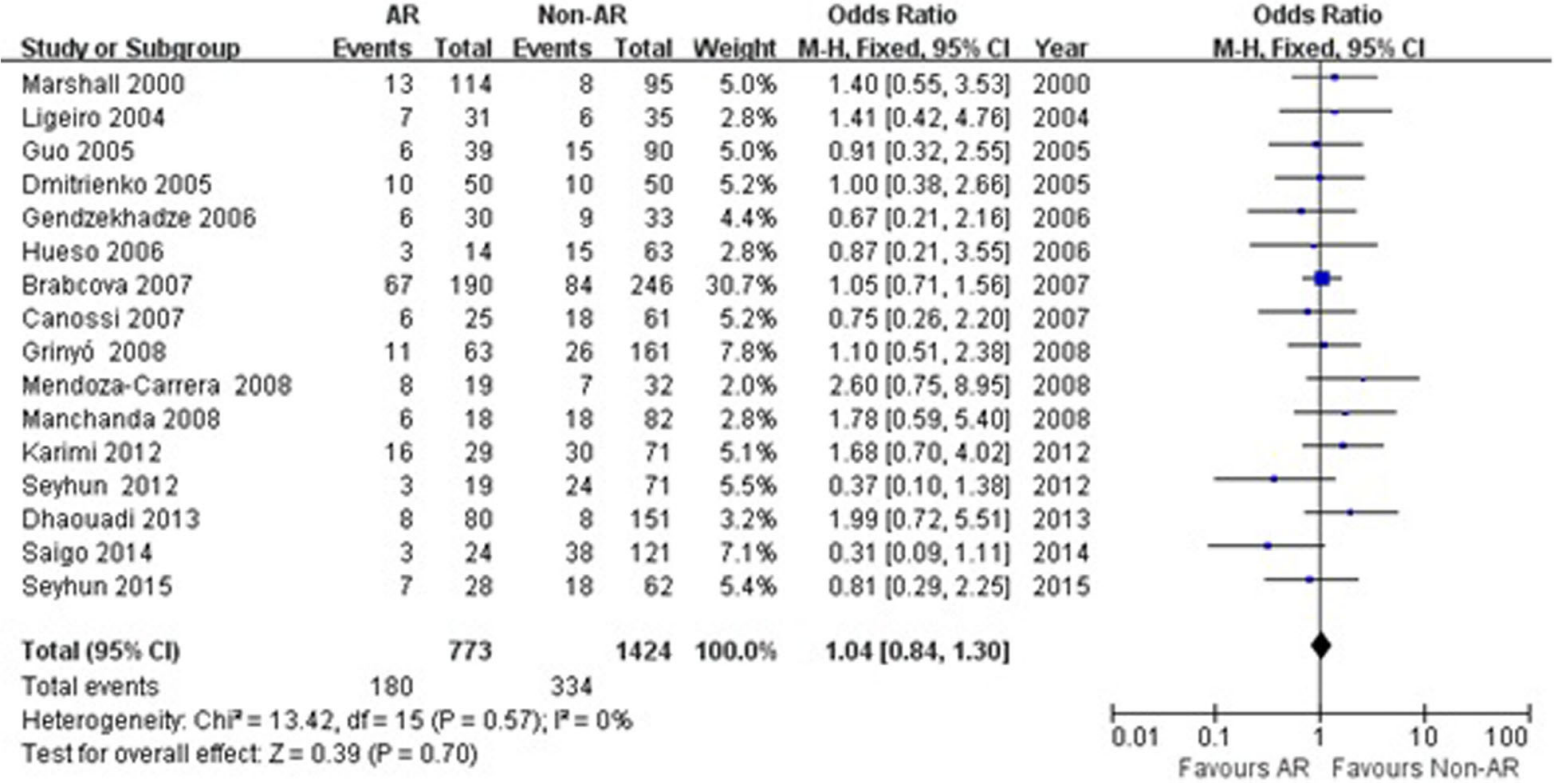
Association between the TGF-β1 + 869 T/C CC genotype and risk of acute rejection in renal transplantation patients. AR: acute rejection; Non-AR: non- acute rejection; M-H: Mantel-Haenszel; CI: confidence interval

**Fig. 3:**
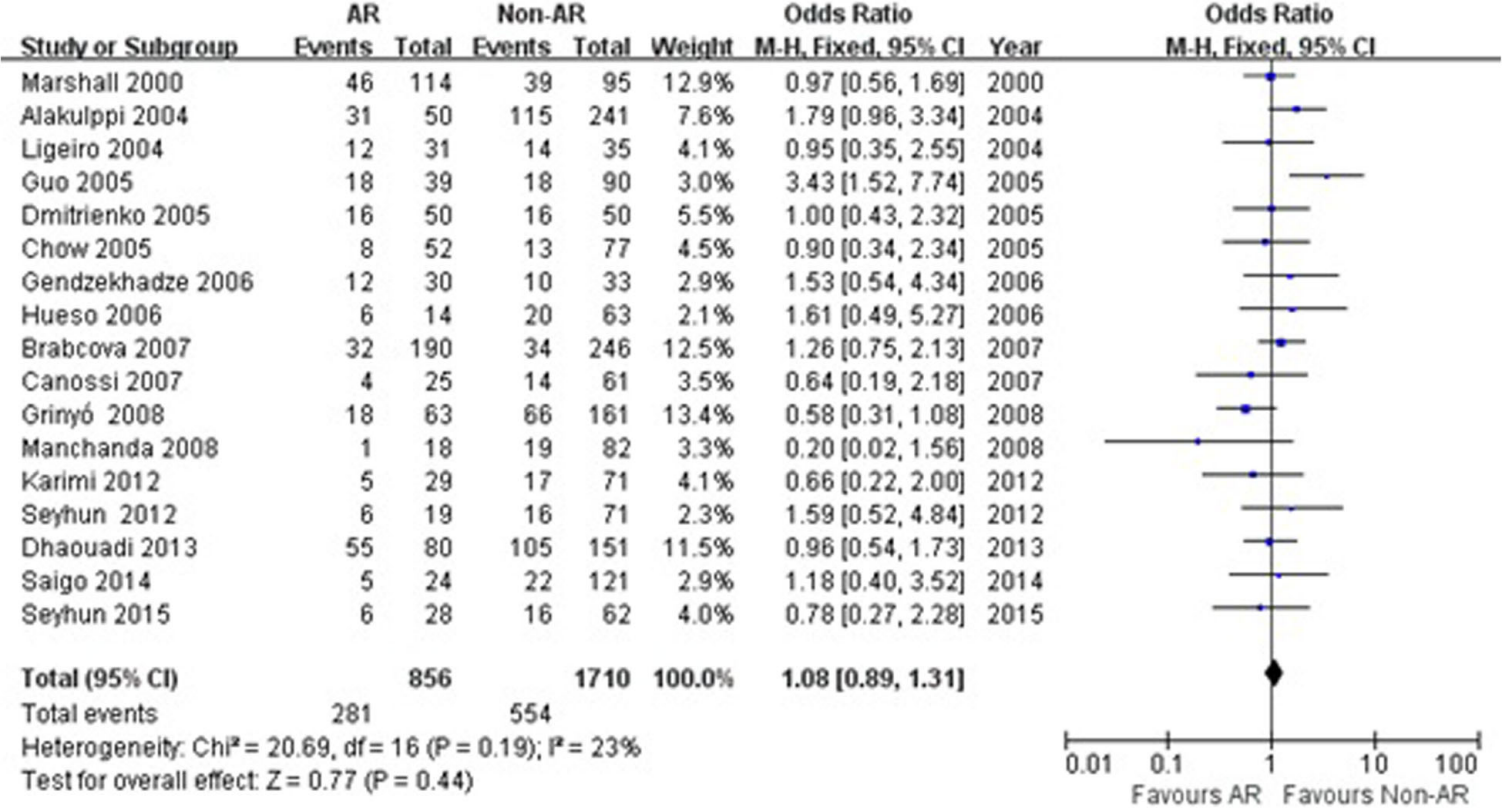
Association between the TGF-β1 + 869 T/C TT genotype and risk of acute rejection in renal transplantation. AR: acute rejection; Non-AR: non- acute rejection; M-H: Mantel-Haenszel; CI: confidence interval

**Fig. 4:**
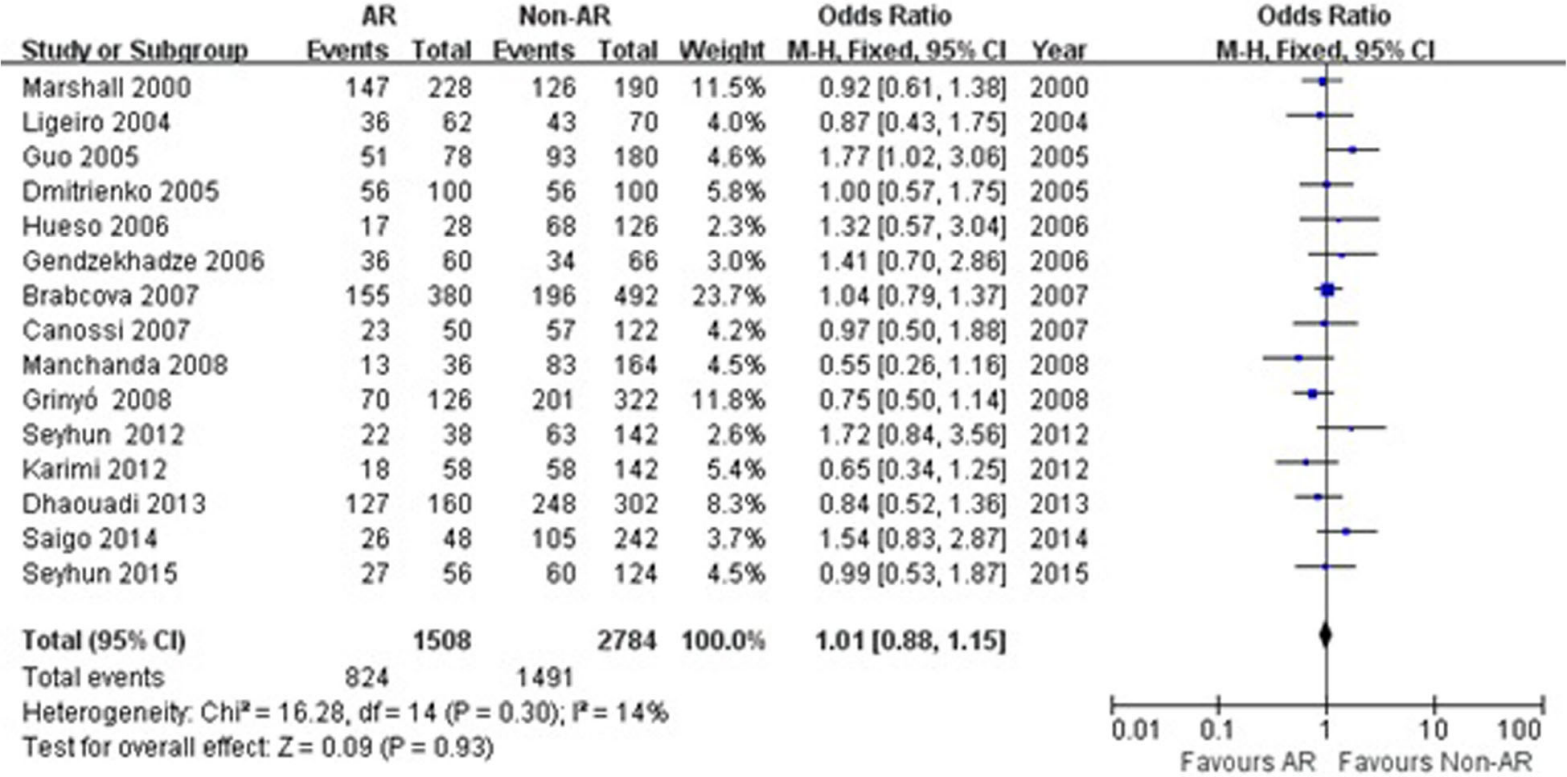
Association between the TGF-β1 + 869 T/C T allele and risk of acute rejection in renal transplantation. AR: acute rejection; Non-AR: non- acute rejection; M-H: Mantel-Haenszel; CI: confidence interval

In this meta-analysis, there was no statistic association between TGF-β1 + 869 T/C gene polymorphism and the susceptibility of acute rejection in patients with renal transplantation (CC genotype: OR = 1.04, 95% CI: 0.84–1.30, *P* = 0.70; TT genotype: OR = 1.08, 95% CI: 0.89–1.31, *P* = 0.44; T allele: OR = 1.01, 95% CI: 0.88–1.15, *P* = 0.93; Fig. 2 for CC genotype, Fig. 3 for TT genotype and Fig. 4 for T allele; Table 3).

**Table 3:** Meta-analysis of the association between the TGF-β1 + 869 T/C gene polymorphism with the risk of acute rejection in renal transplantation

Genetic contrasts	Groups and subgroups	Studies	Q test	Model selected	OR (95% CI)	*P*
			*P* value			
T vs C	Overall	15	0.30	Fixed	1.01 (0.88, 1.15)	0.93
	Caucasian	9	0.78	Fixed	0.99 (0.84, 1.16)	0.88
	Asian	4	0.02	Random	1.03 (0.58, 1.82)	0.93
	African	1	–	Fixed	0.84 (0.52, 1.36)	0.47
TT vs (TC + CC)	Overall	17	0.19	Fixed	1.08 (0.89, 1.31)	0.44
	Caucasian	10	0.45	Fixed	1.05 (0.83, 1.33)	0.67
	Asian	5	0.03	Random	1.06 (0.47, 2.41)	0.88
	African	1	–	Fixed	0.96 (0.54, 1.73)	0.90
CC vs (TC + TT)	Overall	16	0.57	Fixed	1.04 (0.84, 1.30)	0.70
	Caucasian	9	0.89	Fixed	0.99 (0.76, 1.29)	0.95
	Asian	4	0.13	Fixed	1.01 (0.62, 1.67)	0.96
	African	1	–	Fixed	1.99 (0.72, 5.51)	0.19

Then we tried a sub-group to control confounding factor, and the results showed no statistic association between TGF-β1 + 869 T/C gene polymorphism and acute rejection in recipients with renal transplantation in Caucasians, Asians, or Africans as well (Table 3).

### Relationship between TGF-β1 + 915 G/C gene polymorphism and the susceptibility of acute rejection in patients with renal transplantation

**Fig. 5:**
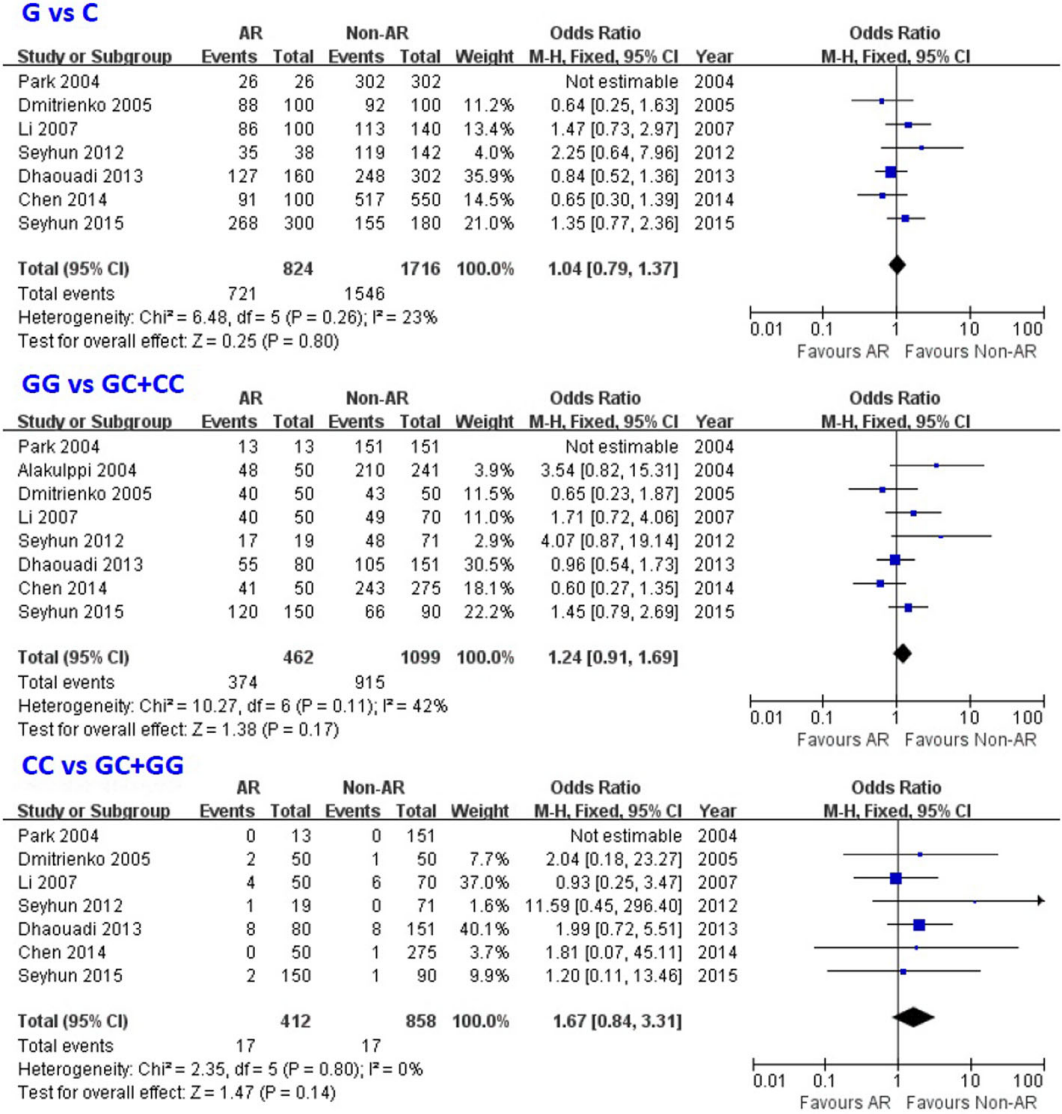
Association between the TGF-β1 + 915 G/C gene polymorphism and risk of acute rejection in renal transplantation patients for overall populations. AR: acute rejection; Non-AR: non- acute rejection; M-H: Mantel-Haenszel; CI: confidence interval

In our meta-analysis, TGF-β1 + 915 G/C gene polymorphism showed no statistic association with the susceptibility of acute rejection in patient with kidney transplantation (CC genotype: OR = 1.67, 95% CI: 0.84–3.31, *P* = 0.14; GG genotype: OR = 1.24, 95% CI: 0.91–1.69, *P* = 0.17; G allele: OR = 1.04, 95% CI: 0.79–1.37, *P* = 0.80; Fig. 5; Table 4).

**Table 4:** Meta-analysis of the association between the TGF-β1 + 915G/C gene polymorphism with acute rejection risk in renal transplantation

Genetic contrasts	Groups and subgroups	Number of studies	Q test	Model selected	OR (95% CI)	*P*
			*P* value			
G vs C	Overall	7	0.26	Fixed	1.04 (0.79, 1.37)	0.80
	Caucasian	4	0.19	Fixed	1.06 (0.72, 1.56)	0.76
	Asian	2	–	Fixed	1.47 (0.73, 2.97)	0.29
	African	1	–	Fixed	0.84 (0.52, 1.36)	0.47
GG vs (GC + CC)	Overall	8	0.11	Fixed	1.24 (0.91, 1.69)	0.17
	Caucasian	5	0.06	Random	1.29 (0.65, 2.55)	0.47
	Asian	2	–	Fixed	1.71 (0.72, 4.06)	0.22
	African	1	–	Fixed	0.96 (0.54, 1.73)	0.90
CC vs (GC + GG)	Overall	7	0.80	Fixed	1.67 (0.84, 3.31)	0.14
	Caucasian	4	0.74	Fixed	2.31 (0.60, 8.89)	0.22
	Asian	2	–	Fixed	0.93 (0.25, 3.47)	0.91
	African	1	–	Fixed	1.99 (0.72, 5.51)	0.19

In the sub-group analysis by ethnicity subsequently, no statistic association was showed between TGF-β1 + 915 G/C gene polymorphism and the susceptibility of acute rejection in patients with kidney transplantation in Asians, Caucasians, and Africans either (Table 4).

### Sensitivity analysis

These studies in HWE were included for sensitivity analysis, and the results indicated that TGF-β1 + 869 T/C gene polymorphism was not associated with the susceptibility of acute rejection in patients with renal transplantation (CC genotype: OR = 0.98, 95% CI: 0.77–1.25, *P* = 0.88; TT genotype: OR = 0.96, 95% CI: 0.77–1.20, *P* = 0.70; T allele: OR = 0.99, 95% CI: 0.86–1.14, P = 0.88). TGF-β1 + 915 G/C gene polymorphism was also not associated with the susceptibility of acute rejection in patient with kidney transplantation (CC genotype: OR = 1.64, 95% CI: 0.80–3.34, *P* = 0.17; GG genotype: OR = 1.22, 95% CI: 0.87–1.70, *P* = 0.25; G allele: OR = 1.09, 95% CI: 0.81–1.45, *P* = 0.58).

We also conducted the sensitivity analysis by omitting each individual study, and found the results were similar to those non-sensitivity analyses. Sensitivity analysis by switching from fixed effect to random effect was also performed and the results indicated that the results were also similar to those non-sensitivity analyses.

### Evaluation of publication bias

**Fig. 6:**
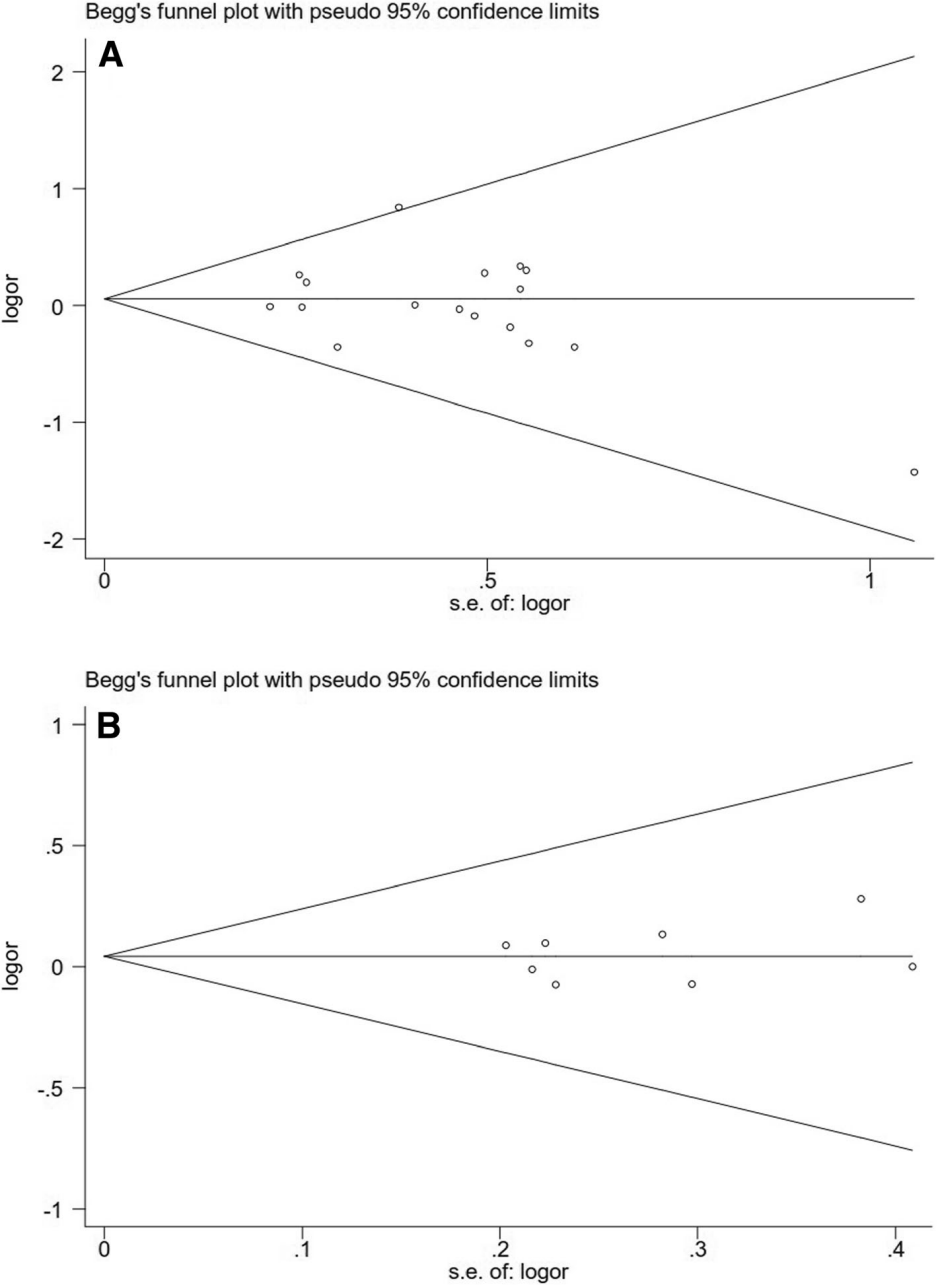
Publication bias was evaluated for the overall populations (**a**: + 869 T/C; **b**: + 915 G/C). OR, odds ratio; SE, standard error

There was no significant publication bias for TGF-β1 + T869C gene polymorphism in overall population (Begg *P* = 0.202, funnel plot was presented in Fig. 6; Egger *P* = 0.420).

No statistic significant publication bias was detected for TGF-β1 + 915 G/C gene polymorphism in the overall populations (Begg *P* = 0.711, funnel plot was presented in Fig. 6; Egger *P* = 0.572).

## Discussion

Some reports [56, 57] showed gene polymorphisms were the susceptibility factor of acute rejection in patients with renal transplantation. However, our meta-analysis results indicated that there were no association between TGF-β1 + 869 T/C gene polymorphism, TGF-β1 + 915 G/C gene polymorphism and the susceptibility of acute rejection in patients with kidney transplantation in the overall population; this relationship was somewhat robust. No publication bias was detected for this analysis, and the results might be robust. Sub-group analysis according to ethnicity was conducted to assess the conclusion.

No association was also found between the TGF-β1 + 869 T/C gene polymorphism, the TGF-β1 + 915 G/C gene polymorphism, and the susceptibility of acute rejection in patients with kidney transplantation in Caucasians, Asians, and Africans in subsequent sub-group analysis. Caucasians, Asians, and Africans were included in those studies but all with small sample sizes. The results also should be regarded cautiously, and more association studies were still needed to assess this relationship further.

The sensitivity analyses were conducted according to HWE and by omitting each individual study and switching from fixed effect to random effect, and the results were similar with those from non-sensitivity analyses. All included studies for this meta-analysis were judged as high quality, so we did not conduct the sensitivity analysis according to NOS score. The results might be robust to some extent, but more association studies were also needed to assess this relationship further.

In previous work, Ge’s et al. meta-analysis of 12 investigations indicated no association in TGF-β1 + 869 T/C gene polymorphism and the susceptibility of acute rejection in patients with renal transplantation in the overall population of China [28]. In our meta-analysis, 18 investigations were included, rendering the sample size much larger, so our results were more robust in some way. Besides, there was no other meta-analysis assessed this relationship, and our results indicated no association between the TGF-β1 + 915 G/C gene polymorphism and the susceptibility of acute rejection in patients with kidney transplantation in Asians, Caucasians, Africans, and the overall population. Nevertheless, these discoveries were considered to be retained, for the reason that many other factors, like objective probability of small sample in the recruited reports, unbalanced cases number between acute rejection group and non-acute rejection group, language bias, limited statistical power, heterogeneity of the enrolled patients, and clinical diversity of different study design and diverse intervention (such as immunosuppressive therapy), are capable of affecting the results. Furthermore, haplotypes analysis might give new information.

## Conclusion

In conclusion, our meta-analysis supported no association between TGF-β1 + 869 T/C gene polymorphism and the TGF-β1 + 915 G/C gene polymorphism with the susceptibility of acute rejection in patients with renal transplantation in Asians, Caucasians, Africans, or the overall human population. However, more association investigations are needed to absolutely justify this verdict.

## Data Availability

The datasets used and/or analyzed during the current study are available from the corresponding author on reasonable request.

## Abbreviations

CKD: chronic kidney disease
ESRD: End-stage renal disease
HD: hemodialysis
PD: peritoneal dialysis
TGF-β1: transforming growth factor-beta 1
